# Active ageing – perspectives on health, participation, and security among older adults in northeastern Thailand – a qualitative study

**DOI:** 10.1186/s12877-020-01981-2

**Published:** 2021-01-12

**Authors:** Manothai Wongsala, Els-Marie Anbäcken, Sirpa Rosendahl

**Affiliations:** 1 School of Health, Care and Social Welfare, Malardalen University, Hamngatan 15, Box 325, 631 05 Eskilstuna, Sweden; 2 Associate professor of Gerontological Social Work, Linkoping University, Senior lecturer in Social Work, School of Health, Care and Social Welfare, Malardalen University, Hamngatan 15, Box 325, 631 05 Eskilstuna, Sweden; 3 Phd Gerontology, Associate professor/Senior lecturer in Nursing, School of Health Sciences, University of Skovde, Hogskolevagen, Box 408, S-54 128 Skovde, Sweden

**Keywords:** Active ageing, Health, Participation, Qualitative content analysis, Security, Thailand

## Abstract

**Background:**

Health, participation, and security are the basic pillars of active ageing suggested by the WHO. The concept is used by professionals but requires exploration from the perspectives of older people themselves. This study aims to explore how older adults experience and describe health, participation, and security.

**Methods:**

A qualitative research approach was used based on open-ended interviews with 20 older Thai adults aged 60–92 years. The interviews were analysed using qualitative content analysis.

**Results:**

The results showed perspectives related to experiences of daily life and local culture. Health while ageing, was described as the absence of barriers to continued daily living, combined with a peaceful and adaptive mindset. Participation was described as maintaining social networks and being a formal volunteer, with an emphasis on the “making of merits”, of meaningful activities and being respected. Security was described as manageable living conditions and managing to finalize life well by balancing dependency and independency in relation to children to sustain the traditional value of gratitude between generations**.**

**Conclusions:**

These understandings will support healthy policy planning by providing resources and activities that relate to older Thai adults’ perspectives of health, participation and security and ultimately contribute to a better quality of life.

**Supplementary Information:**

The online version contains supplementary material available at 10.1186/s12877-020-01981-2.

## Background

Population ageing is a phenomenon that occurs in many countries around the world [1]. Similarly, Thailand has an increasingly ageing population where the number of individuals aged 60 years and older has increased from 9.6 to 17.1% in the last 2 years and is expected to increase to 20% by 2021 [2]. Taking care of the older population is an important mission of the community health system to maintain their long life and well-being. Another reason to support the health of this demographic is also economic. Older adults contribute to the workforce through volunteer work and unpaid caregiving. To develop policy to support older people, it is important to ensure that the positive experience of a long life is accompanied by continuing opportunities for quality of life and wellbeing. Quality of life here refers to a sense of well-being, encompassing happiness and satisfaction with life [3]. The quality of life and well-being of older people could also be described as “ageing well”. There are several conceptions related to the term “ageing well” such as successful ageing [4], healthy ageing [5], positive ageing, productive ageing, [6] and active ageing [7]. These concepts are semantically diverse; however, the goal for all of them relates to the potential of ageing [8].

This study was inspired by the concept of active ageing suggested by the WHO, which is defined as “the process of optimizing opportunities for health, participation, and security in order to enhance quality of life as people age” ([9], p.12). Active ageing includes participation in society and receiving protection, security and care [10]. Health, participation, and security are thus the basic pillars of active ageing. The health pillar is based on the prevention of disease and disabilities. The participation pillar includes work, voluntary activities and learning opportunities according to individual needs. The security pillar aims to ensure the protection, safety and dignity of older people by addressing their social, financial and physical security [11]. Active ageing has been used in the global management of ageing populations, gerontological research and popular media [12]. It is a globally central concept and has been defined in various ways within the context of unique cultures and values across different countries and organizations [13].

Although the concept lacks a precise, universally agreed upon definition, the term has been widely used in many countries in the past 10 years [8]. European countries designated 2012 as the European Year for Active Ageing to raise awareness of the position of older people still playing an active role in society [14]. Many European countries, such as Malta (2014–2020), Ireland (2013), Latvia, Lithuania, and Northern Ireland (2016–2021), have developed and expanded strategic active ageing policies over the past decade [15].

Studies conducted by Bowling [16], Stenner et al. [17] and Marsillas et al. [18] found that older people’s perceptions of the term active ageing included maintaining physical and mental functioning, social activities, and life satisfaction in old age. In a Thai study, older adults identified active ageing as the process of being actively engaged in life in the sense that elderly people made contributions and achieved happiness by doing things that are beneficial for themselves, family and society [19]. Although earlier studies illustrated the subjective perspectives of active ageing, a problem was discerned in regard to the meaning of the term active ageing. Researchers and older adults may use the same word with different understandings. A researcher may understand active ageing as a term that includes several determinants while older people may view the term as two separate words, as active and ageing in a literal sense. For example, a study conducted in England addressed the subjective aspects of active ageing. Stenner et al. [17] found that older people were interested in the word active*,* as indicated by their description of active ageing in terms of activity versus passivity. Active ageing was defined as being alert, being active, or becoming active, which are all opposites of passive ageing. The current study claims that the term active ageing is very difficult to translate into the Thai language in a way that makes sense for people in general. Asking subjects about active ageing in a direct way may risk an invalid understanding of active ageing between researchers and participants. A Thai study [19] showed that even when the concepts of health, participation and security were used in the interviews, it was the overall meaning of ‘active’ in the term “active ageing” that coloured the answers. It can thus be assumed that sociocultural interpretations must be taken into account.

Since the earlier findings thus display varied views on active ageing, the method of investigation must be scrutinized. This is what the current study aims to show by uniquely inverting the active ageing concept by starting with its pillar concepts. Due to the lack of clarity and coverage, it is difficult to apply this concept to adjust policy and develop activity programmes to promote active ageing. Since the active ageing concept aims to optimize opportunities for health, participation, and security to enhance older people’s quality of life, clarifying these basic pillars is necessary. The findings obtained using this approach could thus be beneficial to policymakers and practitioners in community health in promoting active ageing. Exploring older adults’ perspectives on active ageing by breaking down its pillars could eliminate misunderstandings and confusion about the concept.

## Methods

The aim of this study was to explore how older adults in northeastern Thailand experience and describe active ageing through its basic pillars of health, participation, and security. The study was conducted taking a qualitative design and approach, which has been found to be useful in exploring peoples’ experiences as the goal is to gain a deeper understanding of the studied phenomenon. Qualitative content analysis was used as the method of analysis, which can be applied to a variety of data and to various depths of interpretation [20].

### Setting

This study was conducted in the province of Nakhon Ratchasima, which is one of the 20 provinces in the northeastern region of Thailand (Isan region) with a population of 2.62 million. This region of Thailand has a high diversity of cultures. Although the province is home to a range of different cultures as expressed through language, cuisines, and folk songs, the province is also home to the general Isan culture. The Nakhon Ratchasima Province has both rural and urban areas.

### Participants

The inclusion criteria are related to the methodology of qualitative content analysis, which helps researchers obtain a deeper understanding of the studied phenomenon and addresses a variety of factors, i.e., age, gender, education, occupation, health conditions, communicative skills, marital status, cohabitation with family and area of residence. The participants in this study were aged 60 years or above, both men and women, with various education levels and working experience; they were able to communicate well and were willing to participate in the study. Older persons with severe illness were not included because of the prerequisite of being able to come to the meeting place and understand what they were doing without assistance. Another consideration was that persons with severe illness may emphasize illness, especially while being interviewed by a researcher who they perceive as health personnel.

The participants in the urban area were selected from the member list of a senior club. A club staff member informed us with regard to who was qualified and then selected and contacted those individuals. In rural areas, the name list of qualified persons was provided by village health volunteers and selected by the researcher. In addition, the participants also suggested other older persons who were qualified as participants, in accordance with the snowball technique. The first author contacted the older adults by telephone to ask if they were interested in participating in the study. The presumptive participants received both oral and written information about the study**.**

The participants consisted of eight men and 12 women, aged 60–92, with an average age of 75.05. Fourteen of the participants had a primary school education, and six were high school graduates and above. They consisted of four retired teachers, eight farmers, four housewives, two retired mechanics, a retired banker, and a retired government employee. Of these participants, eight were widowed, and 12 were still married. Nine participants were living with a spouse, six were living with adult children, and five were living alone (See Table 1)*.*

**Table 1:** Participants classified by residence gender, age, education, and occupation

	Area of resident				Total	
	Urban		Rural			
	N	%	N	%	N	%
Gender						
Male	3	30	5	50	8	40
Female	7	70	5	50	12	60
Age						
60–70	4	40	3	30	7	35
Over 70	6	60	7	70	13	65
Education						
Primary school	6	60	8	80	14	70
High school	1	10	1	10	2	10
Bachelor	3	30	1	10	4	20
Occupation						
Former teacher	2	20	1	10	3	15
Farmer	4	40	6	60	10	50
Housewife	3	30	2	20	5	25
Former factory worker	1	10	1	10	2	10
Marital status						
Married	5	50	7	70	12	60
Widowed	5	50	3	30	8	40
Habitational status						
With spouse	5	50	4	40	9	45
With adult children	3	30	3	30	6	30
Living alone	2	20	3	30	5	25

### Data collection

The interview guide consisted of open-ended questions emanating from the three basic pillars of active ageing according to the WHO: health, participation, and security [9]. Examples of the questions asked from each pillar include the following: *How do you think about older persons’ health? How should older persons participate socially, with people, and community? What makes you feel secure in life*? (*See* Additional File 1). The guide was vetted by the study’s authors to ensure internal validity. A pilot study to test the interview questions was conducted with five older persons similar to the prospective participants before the actual study began, and the interview questions were improved. The participants chose where they wanted to be interviewed. Almost all participants were interviewed in their own homes, while three were interviewed at the senior club. The interviews were conducted in Thai and were audio-recorded. Each interview took 30 to 80 min, and the average was approximately 40 min. The recorded interviews were transcribed verbatim, and half of these were translated into English, which was considered sufficient to strengthen the analysis as two of the authors were non-Thai speaking. The first author performed the first draft of the analysis, and the third author was involved in the initial categorization with critical reading. All authors read the first draft of possible categories and discussed them until consensus was reached.

### Data analysis

The method of the data analysis was qualitative content analysis according to Graneheim and Lundman [21]. The first step included reading the interviews several times to obtain a sense of the whole. The second step involved reviewing the data to identify the meaning units that were related to the aim of the study. The third step involved condensing the text, i.e., shortening the text so that the core meaning of the unit remains. In the fourth step, the meaning units were labelled with codes. In the fifth step, the codes that were similar and different from one another were sorted into subcategories. In the sixth step, the subcategories with similar content were abstracted into main categories. Finally, the categories were separately checked and revised. The categories were discussed between the authors until agreement was reached about the final main categories and subcategories.

### Ethical considerations

The participants received oral and written information about the overall purpose and protocols of the study and the time required for participation. The right to withdraw from the study without negative consequences was guaranteed, and the participants were asked to sign a consent form. The names were protected by referring the participants as male/female and age. Confidentiality was maintained in all data collection and analysis processes by keeping the material in a safe place and protecting against exposure. This study was approved by ethical review boards in Sweden and Thailand.

## Results

The results present how older adults in northeastern Thailand experienced and described active ageing, which is based on the pillars of health, participation, and security. The categories and subcategories of the results are presented in Table 2*.*

**Table 2:** Categories and subcategories of findings

Basic Pillars	Main Categories	Sub-categories
Health	Status of physical body	Absence of severe illness and physical pain
		Having enough physical capacity to manage daily life.
	Status of mind	Having peace of mind
		Being able to review life
Participation	Maintaining social network	Joining neighbor events and community activities
		Being a formal volunteer
	Supporting spirituality	Joining religious activities
		Doing useful things for others
Security	Living conditions	Social status
		Economic status
		Emotional status
	Managing to finalize life well	Financial preparations
		Thoughts about dying

### Health

Health was presented in two main categories. First, the *status of the physical body* included two subcategories: absence of severe illness and physical pain and sufficient physical capacity to manage daily life. Second, *status of mind* included two subcategories: having peace of mind and being able to reflect on and come to terms with one’s life. The participants seemed to express their perspectives on health that were related to their experiences as well as the expected health status.

Body status was described as related to physical symptoms and functional capacity as well as having a balanced lifestyle. The participants described ‘health’ as the absence of severe illness, physical pain and being able to manage daily life without physical limitations.

*A healthy person is someone who has no illness, no physical pain, no diabetes, no high blood pressure and no heart disease and should not take medicine. (Female, 66)*

A male participant, on the other hand, stated that ‘health’ does not necessarily mean a complete absence of illness. When a condition is controlled by medication and daily life is not affected by the disease, one might feel healthy.

*I think older adults in general have some chronic diseases; it is not a problem when it’s controlled by medicine or healthy lifestyle. I take medicine to control my blood pressure, and I think I’m healthy. (Male, 67)*

Moreover, a healthy life was characterized by balance in terms of physical exercise and intake of food. According to the participants, balance is not doing too much or too little (“por dee” in Thai) of anything; it is an awareness of, but not being too strict in maintaining, a healthy lifestyle.

Having enough physical capacity to manage daily life was described by most participants. This means that healthy persons are able to take care of themselves, continue with their daily routines and have the same abilities as when they were younger and thereby not be a burden to their family and society. In addition, from a gender perspective, female participants thought of their ability to look after housework and family while male participants focused more on their physical abilities to maintain daily living.

*As I’m in my seventies, I think I’m a strong person. I can work. I feel pain when the weather is cold. I can do housework and take care of my family, just have a rest when I’m tired. (Female, 76)*

*Health is being able to rely on ourselves, still being able to use our body to do everything, such as drive a car, read a book, go to the toilet or meditate for one hour. Everything is the same as we used to do without relying on the others. (Male, 92)*

Furthermore, the participants experienced that mobility limitations are a serious health problem. This results in a lower capacity to take care of oneself, to attend to one’s duties, and to participate in social activities and important personal relationships.

*Status of mind* was described as being able to relax and control one’s thinking. From the participants’ perspective**,** older persons should be less materialistic and ambitious, which results in happiness and is viewed as a way to achieve good mental health. A peaceful state of mind was expressed as the absence of and choice not to worry since that could lead to distress and dissatisfaction. Feelings of both sadness and joy should be expressed, not bottled up.

*Whatever makes you happy or joyful, you should let it out. You should let off the steam from being unhappy and express happiness in whatever way you can. (Male, 82)*

Participants pointed out that expressing happy feelings through action increases the experience of joy. Engaging in activities that make one happy, such as singing or dancing, increases happiness in life. The participants expressed that old age is a time to review life and make changes such as giving up drinking and gambling and doing good deeds (=earn merit).

*Bad things that we used to do like gambling or cockfighting for gambling should stop by now. I keep thinking that at this age, I’ve done everything I have wanted to, and it is time to take a break from all this. It is better to use time to go see friends and earn merit for myself. (Male, 82)*

The participants said that old age is a time to think about your own morals and consider how to interact with people based on self-understanding and how people understand you. Older adults should not be self-centred and harbour anger because it causes distress. However, having an optimistic outlook on life and forgiving people are ways to attain peace of mind and contentment. In summary, older adults in northeastern Thailand described health as a combined status of body and mind. The status of mind seemed to imply a connection to spiritual aspects of life.

### Participation

Participation is presented in two main categories. First, *maintaining a social network* includes two subcategories: joining neighbourhood events and community activities and being a formal volunteer. Second, *supporting spirituality* includes two subcategories: joining religious activities and doing useful things for others. Maintaining a social network is an important form of participation and a part of the participants’ well-being. The participants said that maintaining a social network is crucial. Joining neighbourhood events and participating in community activities such as weddings, ordinations and new house ceremonies are important. The participants emphasized that joining in others’ events and expecting them to respond in similar ways is how one can maintain relationships. Joining community activities is an important way to remain part of society. Being socially involved with friends and society results in life satisfaction and longevity.

*When neighbours have weddings or Buddhist ordination ceremonies at their home, I have to join and donate some money. If I don’t join their events, they ignore me when it is my turn (Female, 75)*

*We’re helping to make a better community. I do everything because people know me. People greet me whenever we meet. It makes me happy. (Male 84)*

Taking the role of a volunteer is an activity that makes older adults feel accepted and respected by members of society. There are many kinds of formal volunteers in northeastern Thai society such as the Village Health Volunteer, which was established by the Ministry of Public Health, and other formal groupings established by various government organizations. Being a formal volunteer was associated by the participants with being a helpful and amiable person who is capable of maintaining a social network.

*A health provider invited me to be a village health volunteer (VHV). I have been working full-time. It is very important for my life. I feel ‘very happy’! I would recommend someone else to follow [this path], especially my son. It’s good for me to do this for others. (Female, 67)*

The participants expressed that good participation for them is also related to spirituality. Supporting spirituality was described as joining religious activities and doing useful things for others.

*I go to the temple every day. When I wake up in the morning, I set a pot of rice, do some work, then prepare rice and foods for monks at the temple. I usually earn merit by donating money to the temple. (Female, 79)*

Some of the older adults said that they have less time remaining to accumulate merit before death; therefore, later life is an important time to accelerate merit-making. This involves not only doing rituals but also making donations and doing good things for other people. Consequently, being a generous person is a way to support spirituality. Doing useful things for others, such as helping people, could create the feeling of being a valuable person by bringing happiness and good things to their life.

*When we have good health, we can benefit the family, the community and the world. I think, I have done the best of my ability. Doing useful things results in happiness like being in heaven. (Male, 92)*

In addition, having a role in the family, such as housekeeping, cooking and taking care of grandchildren, was suggested by the participants who lived with their adult children. This makes them feel valuable instead of burdensome. Positive participation not only benefits one’s social status but also supports the spirituality of the participants.

### Security

Security was described in two main categories. First, *living conditions* included three subcategories: social status, economic status, and emotional status. Second, *managing to finalize life well* includes two subcategories: financial preparations and thoughts about dying.

Living conditions from the perspective of social “status” were described as having good children who take care of their ageing parents, economic status was described as having one’s own money or assets and being supported by the family, and emotional status was described as self-satisfaction and pride. According to the participants, older people should not live alone without relatives and that the support of one’s adult children is important and should be accepted when needed.

*I think children or grandchildren are needed. We need help when we are old or sick... I don’t know how to say. I have three adult children; some are living close to me but some far away. Those who live close always take care of me, while the far away ones send me money every month. Now I live with a son without difficulty. I have money and children as caregivers. (Male, 71)*

The participants expressed that receiving gratitude from children who take care of them or support their living makes the participants proud and adds to their sense of dignity.

Another living factor was economic status, which was described in terms of housing, food and money. Housing and food were not difficult for the participants because they had their own houses or lived as extended families with adult children. In addition, older Thai adults have their own small pensions when retired or are senior citizens with allowances from the government.

*It is enough, 700 Baht [allowance] is enough for me, I don’t use too much. The money sometimes does not last if there were too many events to pay in that month. I always donate to earn merit. For neighbour events, I put money in the invitation envelope they gave me, much or less depending on how much money I have. (Female, 79)*

However, some of the participants complained that sometimes money does not suffice if there are too many ritual donations and neighbour events, so sometimes they skip a donation.

Emotional status was mentioned in terms of feelings of self-satisfaction and stability of mind.

*I feel it is enough for me. I don’t need to chase anything, both good and bad things. I don’t mind praising or reprimanding, but I don’t refuse the good things; I’m just not too concerned about it. It is balance. (Male, 92)*

Moreover, being successful in developing a good life in old age for themselves makes the participants proud, as does the sense of being good parents. The participants voiced that it gives them a feeling of emotional security. The older adults described security from the point of view of living conditions as the first main category. In addition, when discussing life security, many of the participants spoke of death and the preparations they had made. Managing to finalize life well was mentioned in terms of preparing for their impending death, which was comprised of financial measures and preparing the mind by viewing death as something normal and not something to be afraid of. Financial preparations were described as securing money for the funeral. The most common way was to be a member of a funeral fund arranged by the community or a government organization. The participants emphasized concern about the funeral as it is the last time of being treated with dignity as a human being.

*Currently, I am happy, no trouble, no debt. I also have my own funeral fund in my Agriculture Bank Funeral. I live with my children, doing just some things I prefer. I sometimes make some desserts to sell in the flea market. It’s just a hobby (Female, 80)*

Transferring inheritance to children was mentioned by several participants. They explained that the transfer of inheritance not only supported the children’s economy but also reduced worry about inheritance disputes between siblings. Thoughts about dying were mentioned by almost all participants. The thought of dying in the future could be accepted since the older participants expressed that they had done many worthwhile things and completed their mission as parents and human beings.

*I’m ready for death. I’m not afraid of anything. It isn’t difficult for me to think about death. I have done many worthwhile things in my life. Some younger ones have already gone. I’ve helped my children and grandchildren many times. (Female, 76)*

Furthermore, the participants said that dying is something that all must face. Another reason that makes some of the older adults not afraid of dying is earning merit. That means doing good things so that one does not have to worry about life after death.

*I am what I am. I usually earn merit, and I am generally kind. I let my health be as it is. I’m not afraid of death. I’m afraid of being tortured (laugh). I’m not afraid of other things. (Male, 84)*

Preparing for death is important for older people in Thailand, who emphasize that appropriate thinking about death reduces worry both in current life and for life after death. The participants described security from the perspective of living status and managing to finalize life well. Although they seem to regard security as a good living status, they do not disregard the spiritual issues linked to death and dying.

## Discussion

This study shows nuanced perspectives of active ageing in older adults. While earlier studies tend to use the term ‘active ageing’ as a governing concept, the current study focuses on its basic pillars of health of, participation and security. Regarding ‘health’, physically healthy persons were not only considered to be those with less illness or with the capacity to be independent but also those who had fewer barriers to living well such as physical pain and mobility limits. Staying active in maintaining social networks and fulfilling the duties of merit-making was very important to these older Thai people; however, such activities sometimes caused economic burdens. The results also highlighted that older persons needed to join meaningful activities and receive respect. Important aspects of security included obtaining balance between being burdensome parents and maintaining dignity by receiving care from their children. These findings thus have implications on the practice, policy and research levels as will be shown in the following.

The references to physical and mental health in the present study differed from the Thai study by Thanakwang et al. [19], who described physical health as the absence of chronic disease. In contrast, the current study showed that chronic disease was accepted when it was kept under control or did not present a barrier to well-being. The ability to maintain daily function was mentioned by the participants in line with the study by Ferreira et al. [21], which reported that maintaining functional independence is the first step of active ageing and thus improves older ‘adults’ quality of life. The important difference in the current study was that the older Thai participants emphasized the ability to be physically mobile as an indicator of good physical health. The current study found that the capacity of age-related role adaptation is an indicator of mental health. The participants explained that according to their culture, older persons who cannot improve their behaviours or adapt to appropriate age roles are seen by others as displaying poor mental health. Health, in the British study by Bowling [16], was described as a part of active ageing both as the present state of health through investing in and preparing for a long healthy life by engaging in physical activities/sports. The Thai participants in this study associated health with their present health status and were able to manage daily life without physical and psychological limitations but did not consciously invest in activities for maintaining physical health for years to come. Instead, they focused on the existential aspect of health as the Thai participants prepared themselves for life after death, engaging in volunteer work for the making of merit or finalizing life well. In contrast to Bowling [16], the British participants invested in maintaining good health and function in this life while the Thai participants invested in their health from an existential point of view but in doing so remained active in their daily lives.

This study added several reasons for participation related to religion and local traditions and clarified that appropriate participation results in feelings of being part of society, being respected and being regarded as a valuable person. This is different than being involved in leisure and social activities with regard to active ageing as described in Stenner et al. [17]. According to Voraroon et al. [22], older persons felt valued if they had meaningful duties assigned by the staff when they participated in activities, and they were bored, disinterested and frustrated when the activities were meaningless, without duty, and did not match their needs. Regarding this issue, it is therefore necessary to provide meaningful activities for older adults.

Good participation took into account both external motives by joining activities with others to maintain a social network and internal motives as a personal effort to live well. Spirituality can similarly be perceived as doing good for others driven by internal motivations; however, it also results in being accepted by other people in the community. Older persons who needed appreciation and respect from children, grandchildren, and friends were also found in a Thai study by Manasatchakun [23]. In late life, they mention having less time to accumulate merit that benefits current living and the next life. Joining events and earning merit were important; however, since some events cost money, which may cause difficulties for older adults with low economic status. The challenge is how to provide opportunities to participate in activities that are not too expensive and still provide a feeling of self-esteem. The current study found a noteworthy contrasting aspect of taking care of older parents in Thailand. Although being taken care of by adult children may result in feelings of being a burden, it was also proof of successful parenting, resulting in feelings of pride. When the children are dutiful, the family will be admired, and the parents will be perceived as successful in their parenting. In the pillar of participation, the responses from the Thai participants and the European older adults were somewhat similar in the sense of engaging in and maintaining social networks. While the older Thai adults were actively engaged in their families, relatives and the local community, the British older adults in the study by Bowling [16] were engaged in social activities locally but also further away geographically, i.e., travelling and moving abroad.

The perspectives on security were described from social and economic perspectives as influenced by others as well as on an emotional level, i.e., self-satisfaction and stability of mind. Consistent with Buddhist belief, older people acknowledge and learn to accept that all things are uncertain; thus, they avoid feeling worried and try to be at peace [24]. The results of this study showed that important perspectives on security could emanate from these feelings of older adults. The participants trusted merit-making as a means to safeguard security after death and that dying well and having a good funeral means dignity for them and their family. Thus, preparedness for death is an important topic for older Thai people in terms of security. In the pillar of security, the living and financial situation was mentioned as important to the Thai participants as well as the British older adults in the study by Bowling [16]. However, the Thai participants were more focused on aspects of balancing dependency and independency in relation to their children to sustain traditional values of gratitude between generations.

By breaking down the term active ageing to its three basic pillars and clarifying the subjective perspectives of older adults, certain misunderstandings regarding the concept of active ageing found in earlier studies have been disentangled. Moreover, a more nuanced understanding of active ageing emerged. Studying health, participation, and security separately elicited perspectives on ageing well. For example, asking about ‘health’ could support the collection of information related to ‘health’ whereby the same meaning is shared by the researcher and the participants. Exploring each pillar separately could yield more detailed perspectives of ‘active ‘ageing’, going beyond the image of being active and focusing more on ageing well. This approach more concretely showed the benefits of such active ageing on a practical level. The results illustrated that there are diverse perspectives on ageing well based on individual experiences. The study clarified the participants’ views on the characteristics of healthy older adults, the desire to be social participants in the community and what they need to feel secure. Their main goals are to maintain the capacity to continue daily living without too many barriers, to participate in activities that they find meaningful to have a good life in old age characterized by dignity until the end of life. Knowledge from this study could constitute a practical guideline to develop policies and practices for ageing well for older people in northeastern Thailand.

Limitations: Most of the participants described themselves as healthy and living well. The results may lack the perspectives of older adults with serious chronic illness, disability and low living status as well as those who are single or have no children. Although this study mentioned cohabitational status and marital status, we did not emphasize exploration of the effect of these characteristics, and the results may also lack a difference in perspectives influenced by these factors. However, the results here did not show distinct differences. The setting was in a province that may not be representative of all older adults in northeastern Thailand. Suggestions for further studies could therefore be to include other categories of older adults and other areas of Thailand.

## Conclusions

The understandings obtained from this study can assist in healthy policy planning by providing resources and activities that relate to older Thai adults’ perspectives of health, participation and security and ultimately contribute to a better quality of life. Living in a globalized world, similarities may appear in terms of the importance of voicing and shaping perspectives on ageing well, even when using common definitions or “pillars” of active ageing: health participation and security. Thus, this study can add insights not only for Thai contexts but also for global applications in public health and preventive care in the community.

### Implications for practice

This study reveals the importance of involving older adults themselves in programmes and activities designed for them. Especially important is to start with their views and build activities from there. Such activities may emanate from their already existing family and community activities while also including new approaches as the views of individuals and families in Thailand undergo changes. This may be more or less a challenge depending on different cultures and contexts. While the study took place in northeastern Thailand, the basic idea can be applied generally in programmes for older people in public health, nursing and community care. It involves hard work to ‘undo’ common ways of planning for activities by health care staff and to take the point of view of older adults. It can similarly be useful in policy work, not least locally, when active ageing policies are to be implemented, to involve older people as cooperation partners to enhance their quality of life.

## Supplementary Information

**Additional file 1.**

## Data Availability

The datasets generated and/or analysed during the current study are not publicly available due to the restrictions of our ethical vettings and as this is an ongoing doctoral research project with unpublished data, but are available from the corresponding author on reasonable request.

## Abbreviations

AAI: The Active Ageing Index
WHO: World Health Organization
