# Supplementary material for: Active ageing – perspectives on health, participation, and security among older adults in northeastern Thailand – a qualitative study
Source: BMC Geriatr. 2021 Jan 12;21:41. doi: 10.1186/s12877-020-01981-2 (PMC7802255; doi:10.1186/s12877-020-01981-2)
Supplement: Supplementary file 1 — Additional file 1. [file 12877_2020_1981_MOESM1_ESM.docx]

*Active Aging - Perspectives on Health, Participation and Security Among Older Adults in North-eastern Thailand – a qualitative study*

***The interview guide.***

1. *How do you think about older persons’ health?*

*Could you please suggest what older persons should do to keep their health?*

*Could you please tell about your health?*

*How do you wish your health to be?*

1. *How should older persons participate socially, with people, and community?*

*Could you please tell about your social participation?*

*Could you please tell about older people’s participation in society?*

1. *Could you please tell about security in life for older persons?*

*What makes you feel secure in life?*

*Do you feel you have a good secure life?*

*What do you do to keep good security in life?*
